# RECRUITMENT OF OLDER ADULTS FOR CLINICAL RESEARCH: STRATEGIES, COSTS, AND REPRESENTATION

**DOI:** 10.1093/geroni/igae098.0404

**Published:** 2024-12-31

**Authors:** Barbara Nicklas, Paul Coen, Jay Magaziner

**Affiliations:** Co-Chair; Co-Chair; Discussant

## Abstract

Clinical research is crucial for identifying new preventative strategies and treatments to address the diverse health care needs of older adults. A necessary component of conducting ethical and unbiased research is to successfully enroll enough study participants who represent the population being studied in a cost-effective and timely manner. Despite the NIH policy mandating that investigators include participants of all ages or specify an acceptable reason for excluding participants based on age, many studies still lack appropriate representation of older adults. Furthermore, an important objective of the NIA’s Strategic Directions for Research for 2020-2025 is to “Develop and implement strategies to increase inclusion of underrepresented populations in aging research”. To implement appropriate strategies for enhancing participation of historically excluded subpopulations, additional data are needed regarding the degree of underrepresentation of these individuals in the research being conducted and reasons for lack of inclusion. This symposium includes three presentations regarding recruitment of older adults for clinical research. These include a compiled analyses of studies conducted over the past 20 years at the Wake Forest OAIC, and data on recruiting older adults for the national Molecular Transducers of Physical Activity Consortium and the two-site observational Study of Muscle, Mobility and Aging. The data presented in the first two talks provide objective observations of the most effective strategies, and the costs, for recruiting older adults. The third presentation compares basic demographics of those enrolled to those who show interest and are screened for participation, and compared to the characteristics of the local population.

